# Supplementary material for: High Diversity and Prevalence of Rickettsial Agents in Rhipicephalus microplus Ticks from Livestock in Karst Landscapes of Southwest China
Source: Microorganisms. 2025 Mar 27;13(4):765. doi: 10.3390/microorganisms13040765 (PMC12029551; doi:10.3390/microorganisms13040765)
Supplement: Supplementary file 1 [file microorganisms-13-00765-s001.zip › microorganisms-3523889-supplementary Figures.pdf]

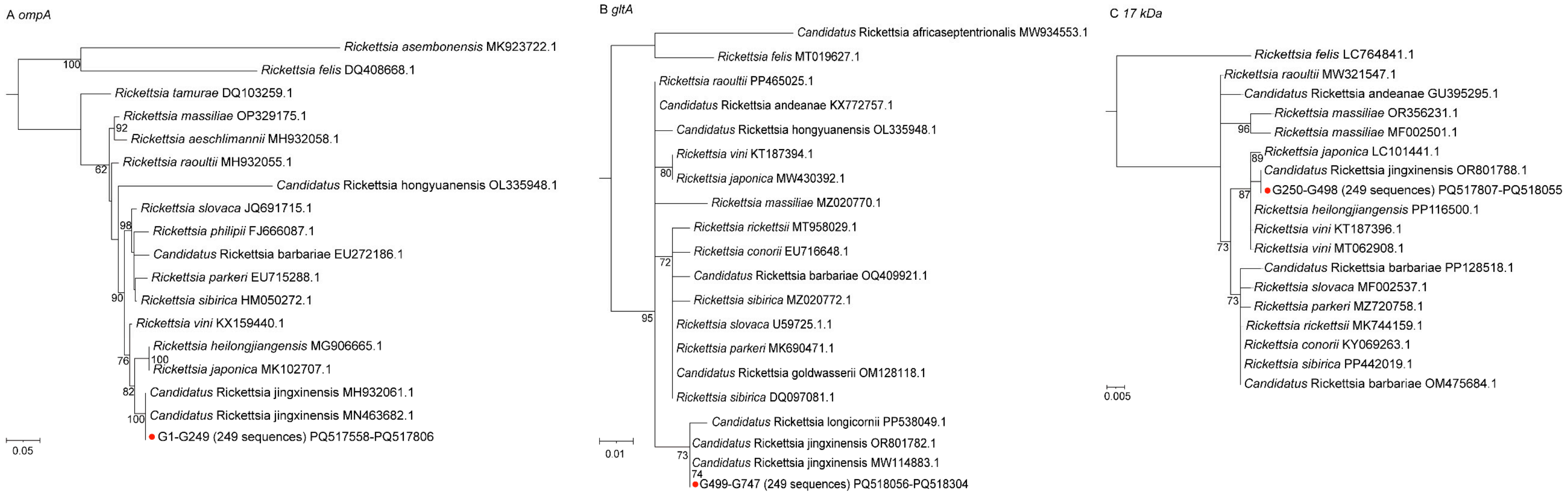

Figure S1. Phylogenetic trees of *Rickettsia* were constructed using the maximum likelihood (ML) method with 1000 bootstrap replicates. (A) *ompA* gene; (B) *gltA* gene; (C) *17 kDa* gene.

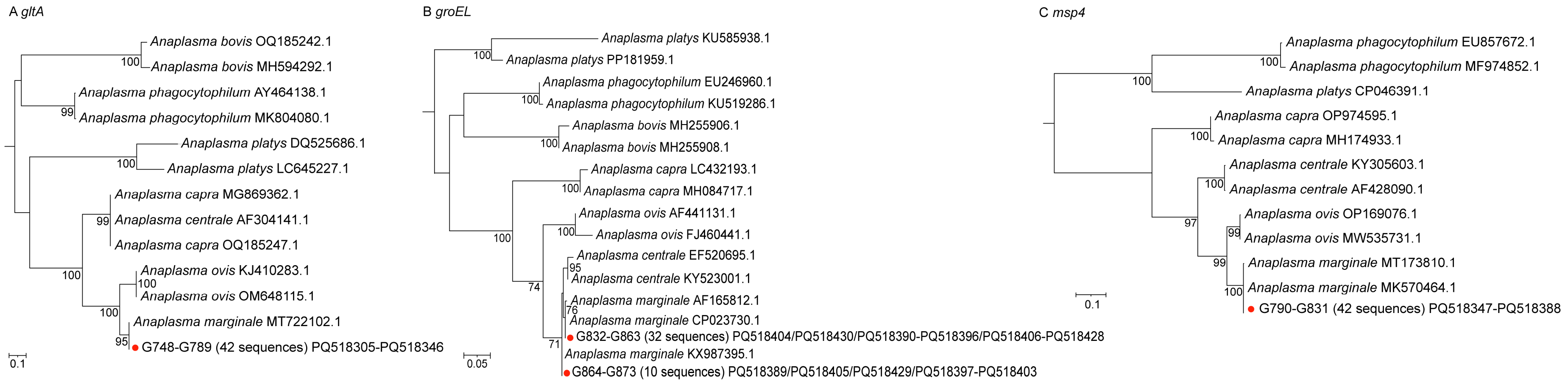

Figure S2. Phylogenetic trees of *Anaplasma* were constructed using the maximum likelihood (ML) method with 1000 bootstrap replicates. (A) *gltA* gene; (B) *groEL* gene; (C) *msp4* gene.

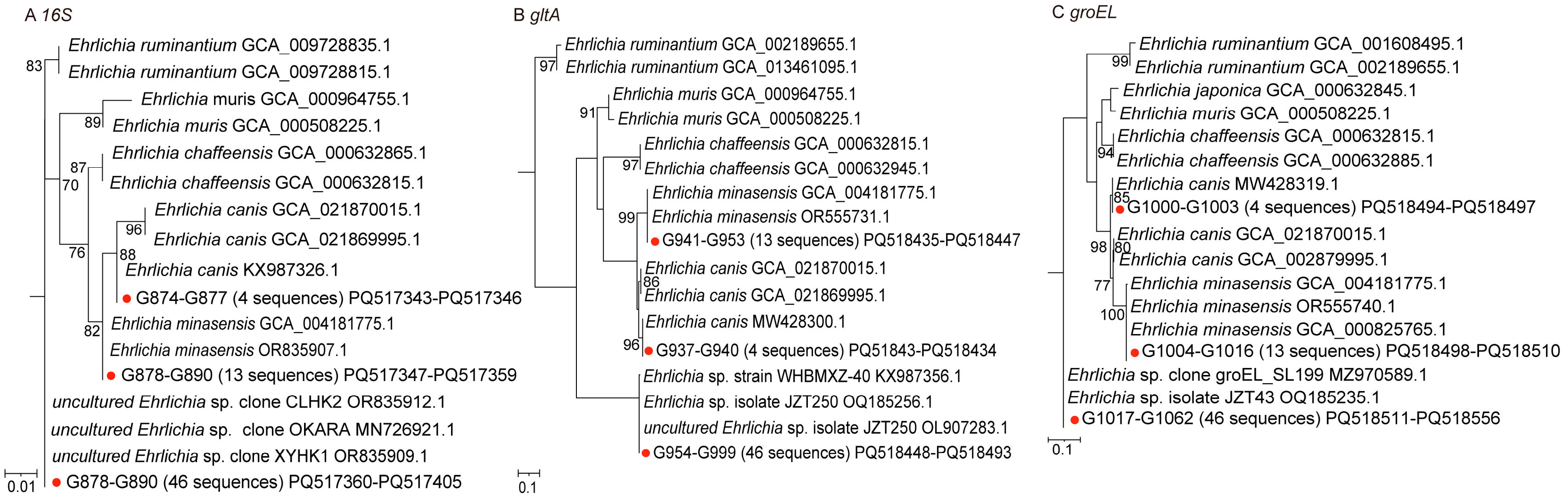

Figure S3. Phylogenetic trees of *Ehrlichia* were constructed using the maximum likelihood (ML) method with 1000 bootstrap replicates. (A) 16S rRNA gene; (B) *gltA* gene; (C) *groEL* gene.
