# Supplementary material for: High Diversity and Prevalence of Rickettsial Agents in Rhipicephalus microplus Ticks from Livestock in Karst Landscapes of Southwest China
Source: Microorganisms. 2025 Mar 27;13(4):765. doi: 10.3390/microorganisms13040765 (PMC12029551; doi:10.3390/microorganisms13040765)
Supplement: Supplementary file 1 [file microorganisms-13-00765-s001.zip › microorganisms-3523889-supplementary Tables.pdf]

Table S1. PCR primers used in this study.

Species	Target	Primer name	Primer sequence (5'–3')	Annealing temperature (°C)	Amplicon size (bp)
Rickettsia spp.	*ompA*	70F	ATGACGAATATTTCTCCAAAA	50	630
		701R	GTTCCGTTAATGGCAGCATCT		
		70F	ATGACGAATATTTCTCCAAAA	50	530
		602R	AGTGCAGCATTCGCTCCCCCT		
	gltA	CS2d	ATGACCAATGAAAATAATAAT	50	1100
		CSEndr	CTTATACTCTCTATGTACA		
		RpCS877f	GGGGACCTGCTCACGGCGG	52	381
		RpCS1258r	ATTGCAAAAAGTACAGTGAACA		
	17kDa	17K3	GCTTTACAAAATTCTAAAACCATATA	52	500
		17k5	TGTCTATCAATTCACAACTTGCC		
		Tara17KD13s1	ATTGTCCGTCAGGTTGGC	52	395
		Tara17KD408r1	CGGGCGGTATGAATAAGC		
Anaplasma spp.	*msp4*	AOmp4-63f	CCTGCTCCCTACTTGTTA	52	706
		AOmp4-769r	GTTATGTGCGGGTATGTC		
		AOmp4-168f	CTTACAGCCCAGCGTTTC	53	597
		AOmp4-765r	TGTGCGGGTATGTCCTTG		
	gltA	Outer–f	GCGATTTTAGAGTGYGGAGATTG	53	1077
		Outer–r	TACAATACCGGAGTAAAAGTCAA		
		Inner–f	GGGTTCMTGTCYACTGCTGCGTG	53	793
		Inner–r	TTGGATCGTARTTCTTGTAGACC		
	groEL	37f	AAATCTATAAGGGAGGTAGTGC	50	1474
		1474r	CGTTAGCGTAGTTCATGGTG		
		37f	AAATCTATAAGGGAGGTAGTGC	50	1000
		1048r	GGCTAGTCCTGCTGGTAAT		
Ehrlichia spp.	16S rRNA	EHR1-out	GAACGAACGCTGGCGGCAAGC	60	691
		EHR2-out	AGTA[T/C]CG[A/G]ACCAGATAGCCGC		
		EHR3-in	TGCATAGGAATCTACCTAGTAG	52	524
		EHR4-in	CTAGGAATTCCGCTATCCTCT		
	gltA	Eh-gltA-112F1	GGRRTRTTAACTTATGATCCAGG	55	575
		Eh-gltA-686R1	GCATTYTGYTCATGATCAGCATG		
		Eh-gltA-137F2	TTATGTCTACTCGCTGCTTGTGA	55	478
		Eh-gltA-614R2	TARGAAGAAAYRTCAAACATCATATG		
	groEL	gro607F	GAAGATGCWGTWGGWTGTACKGC	*	730
		gro1294R	AGMGCTTCWCCTTCWACRTCYTC		
		gro677F	ATTACTCAGAGTGCTTCTCARTG	**	364
		gro1121R	TGCATACCRTCAGTYTTTTCAAC		
Borrelia recurrentis	16S rRNA	Brm1	CGCTGTAAACGATGCACACTTGG TGTTAATC	60	500

h

		Brm2	CGGCAGTCTCGTCTGAGTCCCCATCT		
Borrelia burgdorferi	5S-23S	23S3	CGACCTTCTTCGCCTTAAAGC	55	412
		23Sa	TAAGCTGACTAATACTAATTACCC		
		23S5	CTGCGAGTTCGCGGGAGA	59	253
		23S6	TCCTAGGCATTCACCATA		
Theileria/Babesia	18S rRNA	piroA	AATTACCCAATCCTGACACAG	30	373
		piroB	TAAATACGAATGCCCCCAA		

*This step was set as 10 cycles of 58°C for 30 s and 72°C for 45 s, followed by 25 cycles of 53°C for 30 s and 72°C for 45 s.

**This step was set as 10 cycles of 56°C for 30 s and 72°C for 35 s, followed by 25 cycles of 53°C for 30 s and 72°C for 35 s.

Table S2. Sequences of *Rickettsia*, *Anaplasma*, and *Ehrlichia* deposited in GenBank.

Species	GenBank accession number					
	ompA	***17 kDa***	***gltA***	**16S rRNA**	***groEL***	***msp4***
Candidatus Rickettsia jingxinensis	PQ517558 - PQ517806	PQ517807 - PQ518055	PQ518056 - PQ518304	NA	NA	NA
Anaplasma marginale	NA	NA	PQ518305 - PQ518346	NA	PQ518389 - PQ518430	PQ518347 - PQ518388
Ehrlichia canis	NA	NA	PQ518431 - PQ518434	PQ517343 - PQ517346	PQ518494 - PQ518497	NA
Ehrlichia minasensis	NA	NA	PQ518435 - PQ518447	PQ517347 - PQ517359	PQ518498 - PQ518510	NA
Candidatus Ehrlichia carsus	NA	NA	PQ518448 - PQ518493	PQ517360 - PQ517405	PQ518511 - PQ518556	NA

NA: The targeted gene is not present.

Table S3. Reference sequences utilized for combined phylogenetic analysis.

Species	Strain	Genome (Accession no.)	GenBank Accession no.					
			ompA	*17 KDa*	*gltA*	16S rRNA	*groEL*	*msp4*
Rickettsia bellii	An04	CP015010.1	†	†	†	NA	NA	NA
Rickettsia felis	URRWXCal2	CP000053.1	†	†	†	NA	NA	NA
Candidatus Rickettsia asemboensis	F82	‡	JN315977.1	JN315975.1	JN315974.1	NA	NA	NA
Rickettsia massiliae	AZT80	CP003319.1	†	†	†	NA	NA	NA
Rickettsia raoultii	Khabarovsk	CP010969.1	†	†	†	NA	NA	NA
Rickettsia japonica	Nakase	AP017578.1	†	†	†	NA	NA	NA
Rickettsia philipii	364D	CP003308.1	†	†	†	NA	NA	NA
Rickettsia rickettsii	Taiacu	CP098689.1	†	†	†	NA	NA	NA
Rickettsia africae	ESF-5	CP001612.1	†	†	†	NA	NA	NA
Rickettsia parkeri	Atlantic Rainforest	CP040325.1	†	†	†	NA	NA	NA
Rickettsia slovaca	D-CWPP	CP003375.1	†	†	†	NA	NA	NA
Rickettsia conorii	Malish 7	AE006914.1	†	†	†	NA	NA	NA
Rickettsia sibirica	246	AABW01000001.1	†	†	†	NA	NA	NA
Rickettsia vini	IA-CR	‡	KX159440.1	KX159431.1	KX159434.1	NA	NA	NA
Candidatus Rickettsia jingxinensis	Meixian-Hl-107	‡	MH932061.1	MH932031.1	MH932016.1	NA	NA	NA
Candidatus Rickettsia jingxinensis	MIVX8BNT1	‡	MN463686.1	MN463687.1	MN463685.1	NA	NA	NA
Anaplasma platys	S3	CP046391.1	NA	NA	†	NA	†	†
Anaplasma ovis	Haibei	CP015994.2	NA	NA	†	NA	†	†
Anaplasma phagocytophilum	JM	CP006617.1	NA	NA	†	NA	†	†
Anaplasma capra	BG346	‡	NA	NA	MT721143.1	NA	MT721150.1	MT721148.1

Anaplasma centrale	Israel	CP001759.1	NA	NA	†	NA	†	†
Anaplasma marginale	Dawn	CP006847.1	NA	NA	†	NA	†	†
Anaplasma marginale	Palmeira	CP023730.1	NA	NA	†	NA	†	†
Ehrlichia ruminantium	Springbokfontein4	GCA_013461095.1	NA	NA	†	†	†	NA
Ehrlichia ruminantium	Palm River	GCA_001608495.1	NA	NA	†	†	†	NA
Ehrlichia chaffeensis	Heartland	GCA_000632815.1	NA	NA	†	†	†	NA
Ehrlichia chaffeensis	West Paces	GCA_000632965.1	NA	NA	†	†	†	NA
Ehrlichia japonica	HF	GCA_000632845.1	NA	NA	†	†	†	NA
Ehrlichia muris	EmCRT	GCA_000964755.1	NA	NA	†	†	†	NA
Ehrlichia muris	AS145	GCA_000508225.1	NA	NA	†	†	†	NA
Ehrlichia canis	21-00950-0007	GCA_021869995.1	NA	NA	†	†	†	NA
Ehrlichia canis	20-02677-0001	GCA_021870015.1	NA	NA	†	†	†	NA
Ehrlichia minasensis	B11	GCA_004181775.1	NA	NA	†	†	†	NA
Ehrlichia minasensis	UFMG-EV	GCA_000825765.1	NA	NA	†	†	†	NA

† = Complete genome available in GenBank

‡ = Individual genes sequences available in the GenBank

NA = Gene sequences not utilized in this study
